# A Topological Framework for Interactive Queries on 3D Models in the Web

**DOI:** 10.1155/2014/920985

**Published:** 2014-04-03

**Authors:** Mauro Figueiredo, José I. Rodrigues, Ivo Silvestre, Cristina Veiga-Pires

**Affiliations:** ^1^Centro de Investigação Marinha e Ambiental (CIMA), Portugal; ^2^Instituto Superior de Engenharia, Universidade do Algarve, 8005-139 Faro, Portugal; ^3^Faculdade de Ciências e Tecnologia, Universidade do Algarve, 8005-139 Faro, Portugal

## Abstract

Several technologies exist to create 3D content for the web. With X3D, WebGL, and X3DOM, it is possible to visualize and interact with 3D models in a web browser. Frequently, three-dimensional objects are stored using the X3D file format for the web. However, there is no explicit topological information, which makes it difficult to design fast algorithms for applications that require adjacency and incidence data. This paper presents a new open source toolkit TopTri (Topological model for Triangle meshes) for Web3D servers that builds the topological model for triangular meshes of manifold or nonmanifold models. Web3D client applications using this toolkit make queries to the web server to get adjacent and incidence information of vertices, edges, and faces. This paper shows the application of the topological information to get minimal local points and iso-lines in a 3D mesh in a web browser. As an application, we present also the interactive identification of stalactites in a cave chamber in a 3D web browser. Several tests show that even for large triangular meshes with millions of triangles, the adjacency and incidence information is returned in real time making the presented toolkit appropriate for interactive Web3D applications.

## 1. Introduction

Advances in computer graphics and scanning technologies have resulted in geometric models of complex objects consisting of millions of polygons. In general, 3D objects are approximated by triangular meshes since graphical cards are optimized for triangles.

The ability to visualize and manipulate these 3D models on the Web is promising for many application areas.

The latest HTML5 specification explicitly utilizes X3D for declarative 3D scenes [1]. The X3D file format represents object surfaces by subdividing or approximating them with triangles and storing the geometry of every vertex of the mesh only once. The triangles are then defined by pointers to the vertices that define them. In this way, the triangle mesh is represented by a shared list of vertices and a list of triangles storing pointers for their vertices. This representation is convenient and efficient for many purposes; however, in some domains it proves to be ineffective. There is no explicit topological information which makes it difficult to design and implement fast algorithms to retrieve adjacency and incidence data. For many applications like mesh simplification [2], multiresolution techniques [3], 3D interaction [4], collision detection [5], and many others, a fast access to adjacent vertices, edges, and triangles is an important aspect for the implementation of efficient algorithms and techniques. When the topological information is not available explicitly, searching topological data is time consuming, in particular for large meshes. Therefore, there is a need for models that also store topological adjacency and incidence data as light as possible to maximize performance and minimize the extra memory overhead for Web3D applications. Of course, the more explicit topological information is stored, the more memory is needed and the faster is accessing the required adjacency and incidence information.

This paper presents a new open source TopTri (Available from http://w3.ualg.pt/~mfiguei/toptri.php) toolkit that adds topological data to triangular meshes of manifold or nonmanifold models. This toolkit can be installed on Web3D servers and constitutes a framework to make interactive topological queries on the Web. This paper shows that getting adjacent and incidence information of vertices, edges, and faces from a Web3D server makes the interactive determination of minimal local points and isolines in a 3D mesh in a web browser possible. The use of topological information can be an important tool in the study of karsts and their geomorphological structures as it is shown here with the interactive determination of stalactites from a cave chamber on a 3D Web browser.

The toolkit is implemented with the Python programming language and builds a topological model on the Web server from the triangle geometry mesh. The topological model implements a graph induced from the mesh geometry that explicitly stores adjacency and incidence information of vertices, edges, and faces.

This paper also presents tests executed with several models of different complexities that show real-time performance to get the adjacency and incidence information for models with millions of triangles.

This paper is organized as follows.  Section 2 presents related work. The topological model presented in this paper is supported by the graph theory that is discussed in Section 3.  Section 4 describes the implementation details of TopTri.  Section 5 analyzes the memory cost for the representation of the topological adjacency and incidence information for the TopTri toolkit and compares it with other data structures.  Section 6 presents examples of functionalities that were implemented using the topological framework TopTri installed in the Web3D server side. Performance evaluation of the TopTri toolkit is discussed in Section 7, and conclusions are presented in Section 8.

## 2. Related Work

Polygonal meshes are the most commonly used representations in computer graphics applications. They are mostly important in modelization for real-time and photorealistic graphics. Polygonal meshes are used in geometric systems and applications such as virtual reality, rendering of solids, and surfaces. They represent surfaces by subdividing or approximating them into simpler surfaces, which are called faces [6]. Polygonal meshes are well suited to scan line rendering and represent therefore the method of choice for real-time computer graphics.

Polygonal meshes can be represented in different ways: explicit, pointers to a vertex list, and pointers to an edge list [7]. In the* explicit* representation, each polygon *P* is represented by a list of vertex coordinates *P* = ((*x*_1_, *y*_1_, *z*_1_), (*x*_2_, *y*_2_, *z*_2_),…, (*x*_*n*_, *y*_*n*_, *z*_*n*_)). In this case, vertices shared by different polygons are duplicated. Algorithms that perform operations on a mesh, like mesh editing or mesh decimation algorithms need more information than the mere shape of each separate polygon. For example, when the geometric position of one vertex is modified, this operation not only affects the shape of a single polygon that contains this vertex, but all polygons containing a vertex at the same geometric position should be modified also. With this structure, it is difficult to find all the polygons that share the same vertex, since it requires comparing the coordinate triples of one polygon with those of all other polygons. Additionally, there can be computational roundoff problems and the same vertex can have slightly different coordinate values making that a correct match might never be made. This problem can be solved with the next structure.

The representation of a mesh with* pointers to a vertex list* has each vertex in the mesh stored just once in a vertex list *V* = ((*x*_1_, *y*_1_, *z*_1_),…, (*x*_*n*_, *y*_*n*_, *z*_*n*_)). A polygon is then defined by a list of indexes into the vertex list. For example, the polygon *P* = (2,4, 6) is a triangle defined by the vertices with indices 2, 4, and 6 in the vertex list. With this structure if the geometric position of a vertex is modified, all polygons sharing this common vertex store pointers to the same physical instance and are also updated. In this way, the vertex geometry is specified once and is shared by an arbitrary number of polygons. However, in such a structure, it is, for example, difficult to find polygons that share an edge. This problem is solved by the next representation.

The mesh can also be represented by* pointers to an edge list*. There is a vertex list *V* = ((*x*_1_, *y*_1_, *z*_1_),…, (*x*_*n*_, *y*_*n*_, *z*_*n*_)) like in the previous representation. A polygon is represented by a list of indexes to an edge list *P* = (*E*_1_,…, *E*_*n*_). Each edge is represented only once by the indexes to the two vertices in the vertex list defining the edge and the two polygons to which the edge belongs, *E* = (*V*_1_, *V*_2_, *P*_1_, *P*_2_). In this case, it is easy to find polygons that share an edge since that information is stored explicitly in the model.

However, in none of these three representations it is easy to determine which edges are incident to a vertex, since all edges must be traversed. The main disadvantage of these three structures is that they do not have explicit connectivity information stored in the data structure. For this reason, it is difficult to implement fast algorithms to find adjacency and incidence data. Rapid access to adjacent vertices, edges, and triangles is an important aspect for the implementation of algorithms and techniques such as multiresolution techniques, subdivision surfaces, mesh simplification, and others.

More information can be added explicitly to determine such relationships. Oriented boundary representation structures like the Winged-Edge [8], Half-Edge [6], the Radial-Edge [9], or the AIF (Adjacency and Incidence Framework) [10] store explicit topological data to speed up algorithms finding adjacency and incidence information. For example, the winged-edge representation expands the edge description to include pointers to the two adjoint edges of each polygon and the vertex description includes a pointer to an edge incident on the vertex. The Adjacency and Incidence Framework is a data structure which is not topologically-oriented that explicitly stores the adjacency vertex to edge, edge to face, and the incidence edge to vertex and face to edge relations. Kallmann and Thalmann [11] proposed the Star-Vertex data structure based on the incidence information around a vertex. It is concise, but the retrieval of adjacency and incidence information is slow since there is no explicit information about edges and faces.

Commonly used file formats follow the pointers to a vertex list data structure, such as: VRML (Virtual Reality Modeling Language), PLY (Polygon file format), and X3D file format. In these cases, there is no explicit topological information which makes it difficult to design and implement fast algorithms to retrieve adjacency and incidence data. In this way, it is, for example, very time consuming to find the set of vertices adjacent to a given vertex, because that requires traversing all the faces of the model. Thus, this search algorithm is time consuming, in particular for large meshes.

Triangular meshes structures are commonly used to model 3D objects since the triangle is currently the only geometric structure that is directly supported by computer graphics hardware. The triangular mesh is a geometric data structure that represents the surface of objects by a set of triangles, where each face is represented by three vertices.

Some data structures that also store topological data were designed specifically for triangular meshes as for example, Directed-Edge [12], Tri-Edge [13], Progressive Meshes [14], Progressive Simplicial Complexes (PSC) [15] and CHE (Compact Half-Edge) [16]. The Directed-Edge and the CHE structures trade memory for access time, enabling a balance between the memory usage and the topological data that is stored explicitly in the model, by either storing internal references explicitly or by locally reconstructing them on demand. Progressive Meshes and the Tri-Edge are concise data structures that are triangle based which are slower to find adjacency and incidence for edges. De Floriani and Hui [17] also introduced a data structure for nonmanifold triangular meshes that explicitly stores the adjacency and incidence vertex to vertex, vertex to triangle, triangle to vertex, and triangle to triangle relations. However, access to the adjacency and incidence information associated to edges is slow since edges are not explicitly represented.

The OpenMesh [18] and the Computational Geometry Algorithm Library (CGAL) (http://www.cgal.org) implement a Half-Edge data structure in C++. These libraries are very powerful because of their flexibility and efficiency; however, C++ is not pratical to use in the server side of a Web3D site [19].

## 3. Graphs, Adjacency, and Incidence

A graph *G* = (*V*, *E*) consists of a set of vertices, *V*, and a set of edges, *E*, where each edge *e* ∈ *E* is an unordered pair of vertices. In the present work, graphs are finite and contain neither loops nor multiple edges. We adopted the notation of Wallis [20].

Two vertices* endpoints* of the same edge are said to be* adjacent* to each other. The set of all vertices adjacent to the vertex *u* is denoted by *N*(*u*). The* degree* or* valency k* of a vertex is the number of vertices adjacent to it.

An edge *uv* is said to be* incident* on its both endpoints, *u* and *v*. Two edges are adjacent if they share a common endpoint.

A* path* is a sequence of pairwise adjacent vertices without repetition of any vertex except possibly the first and the last which can be the same. When the first and the last vertices of a path are the same, the* closed path* is also called a* cycle*. The* length* of a path is its number of edges.

**Figure 1:**
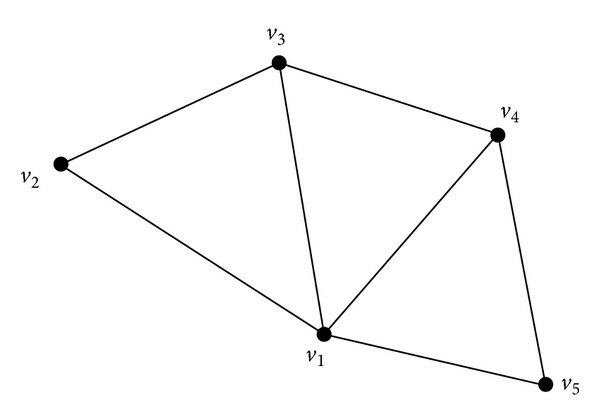
Graph with 5 vertices and 7 edges.

Figure 1 presents a representation of the graph *G* = (*V*, *E*) with 5 vertices (*V* = {*v*_1_,…, *v*_5_}) and 7 edges. Vertex *v*_2_ is adjacent to vertices *v*_1_ and *v*_3_. Edge *v*_3_ *v*_4_ of *E*(*G*) is adjacent to the edge *v*_2_ *v*_3_. The cycle *C* = (*v*_1_, *v*_2_, *v*_3_, *v*_4_, *v*_1_) is of length 4.

The above concepts of adjacent and incidence can be extended to cycles. In this way, a* cycle is incident* on its edges and on its vertices. Two cycles sharing a common edge are* adjacent*.

In this work we are concerned about cycles of length 3 (3-cycles).

In several practical problems it is necessary to identify specific relationships of incidence and adjacency between elements in graphs. Let us see how to query some of these topological relationships. Properties 1, 2, and 3, together, provide the basis for the toolkit implementation presented in Section 4 of this paper.

**Property 1**

Given a vertex *u* ∈ *V*(*G*), then the following properties hold:

- each vertex *v* of *N*(*u*) is adjacent to *u*;
- every edge *uv* for *v* ∈ *N*(*u*) is incident on *u*;
- any existing 3-cycle (*u*, *v*, *w*, *u*) for *v* ∈ *N*(*u*) and *w* ∈ *N*(*u*)∩*N*(*v*) is incident on *u*.

The first and second properties are straightforward consequences of definitions of adjacent vertices and incident edges. The third is also straightforward, pointing that *u*, *v*, and *w* are pairwise adjacent. When *u* and *v* do not share adjacent vertices, such cycle does not exist.

**Property 2**

Given an edge *e* = *uv* ∈ *E*(*G*), then

- each edge* uw* (resp.* vs*) for *w* ∈ *N*(*u*) (*s* ∈ *N*(*v*)) is adjacent to *e*;
- each cycle (*u*, *v*, *w*, *u*) for each *w* ∈ *N*(*u*)∩*N*(*v*) is incident on edge *e*.

**Property 3**

Given the cycle *C* = (*u*, *v*, *w*, *u*) then it is adjacent to the cycles:

- (*u*, *v*, *r*, *u*) for *r* ∈ *N*(*u*)∩*N*(*v*);
- (*v*, *w*, *s*, *v*) for *s* ∈ *N*(*v*)∩*N*(*w*);
- (*u*, *w*, *t*, *u*) for *t* ∈ *N*(*u*)∩*N*(*w*).

## 4. Implementation of the Topological Framework

The topological framework is available as a free toolkit TopTri that implements a graph induced from a triangular mesh. For graph representation, adjacency lists are used, where for each vertex the list of adjacent vertices is stored.

A triangular mesh is a set of triangular faces used to represent or approximate surfaces. Let *G* = (*V*, *E*) be a graph where *v*_*i*_ ∈ *V* if there is a point *P*_*i*_ vertex of a triangle in the mesh in a one-to-one correspondence. That is, there is a bijection between elements of *V*(*G*) and the set of all vertices of the mesh. Vertices *v*_*i*_ and *v*_*j*_ are adjacent (*v*_*i*_ *v*_*j*_ ∈ *E*(*G*)) if the points *P*_*i*_ and *P*_*j*_ are both vertices of one triangular face of the mesh. This definition holds for manifold and nonmanifold meshes.

The toolkit is implemented using* Python* programming language (http://www.python.org). This programming language is a high-level object-oriented language which is very versatile since it can also be compiled (http://www.py2exe.org/), linked with other programming languages (http://www.boost.org/doc/libs/1_36_0/libs/python/

<?tex cmd="noindent"?>doc/index.html) or used in a web server, as we did.

This language includes dictionary and list data structures which allow the user to load graphs data using adjacency lists. Dictionaries, which are hash tables, are mapping objects that map key values to arbitrary objects. Mappings are mutable objects. Dictionaries can be thought as a list of* key: value* pairs where* keys* are usually integer or strings and* values* any object.

One of the advantages of using dictionaries in the implementation of this toolkit is that searching for an element is much faster since it is *O*(1).

The following example shows the adjacency lists of graph in Figure 1:

(1) G={1:[2,3,4,5],2:[1,3],3:[1,2,4],4:[1,3,5],  5:[1,4]}.

In addition to the adjacency lists, the toolkit also includes six methods to achieve topological relationships for vertices, edges, and cycles of length 3. Adjacency methods map each vertex to the list of its adjacent vertices (*vGetAdjacentVertices*), an edge to the list of its adjacent edges (*eGetAdjacentEdges*), and any 3-cycle to the list of its adjacent 3-cycles (*fGetAdjacentFaces*). Incidence methods map each vertex to the list of incident edges and to the list of its incident 3-cycles (*vGetIncidentEdges* and* vGetIncidentFaces*, resp.), and each edge to the list of its incident 3-cycles (eGetIncidentFaces) (see Table 1).

Algorithm 1 shows implementation code in Python of methods* vGetAdjacentVertices* and* eGetIncidentFaces*. The first one,* vGetAdjacentVertices*, is an explicit topological relation; that is, adjacent vertices are retrieved in one single query, and the second,* eGetIncidentFaces*, is an implicit topological relation; that is, incident faces are obtained with two or more queries. Table 1 shows the list of the six implemented methods and the corresponding input. The output is always a list of vertices, edges, or 3-cycles. These methods implement Properties 1, 2 and 3.

Table 2 presents the classification of explicit and implicit methods, which depends on the number of the implemented queries. This table also relates methods with the implemented properties. Properties (a) and (b) in Property 1 depend only on one query (*N*(*u*)), thus* vGetAdjacentVertices* and* vGetIncidentEdges* are explicit methods.

Accordingly, toolkit implements six of the nine topological relationships between vertices, edges, and faces. However, the remaining three were not implemented because they correspond to relations involving the adopted representation of edges and faces. For example, an edge is identified as a pair of vertices (*v*_*i*_, *v*_*j*_), thus relation from edge to vertices is straightforward.

## 5. Memory Cost Analysis

In the explicit representation of a mesh, a triangle is defined by the geometric position of its three vertices. In this way, assuming 4 bytes float values, each 3D triangle requires 3 · 3 · 4 = 36 bytes.

As discussed in Section 2, the representation of a mesh with pointers to a vertex list is the most common way to represent the geometry of 3D models. In such representation, vertices shared by several triangles are stored only once. Consider *n*, *e*, and *f* as the number of vertices, edges, and faces, respectively, then from the Euler's formula *n* − *e* + *f* = 2 [7]. It is known that a manifold triangle mesh (3*f* ≈ 2*e*) with *n* vertices has *f* ≈ 2*n* triangles. These values are confirmed with the three models used to evaluate the performance of the toolkit in Section 7 (see Table 5).

In this way, the representation of a mesh with pointers to a vertex list requires 3 · 4 · *n* = 12*n* bytes to store the geometry of all *n* vertices of the mesh and 3 · 4 · *f* = 12*f* bytes for the triangles pointers to the three vertices, assuming 4 bytes to store each pointer. Therefore, for a triangle mesh with *f* triangles, the total space to store geometry is 12*n* + 12*f* ≈ 18*f* bytes. Compared to the explicit representation of a triangle mesh, the representation of a mesh with pointers to a vertex list saves about fifty percent of the memory amount.

If the triangle mesh is represented by pointers to an edge list, it also requires 3 · 4 · *n* = 12*n* = 6*f* bytes to store the geometry of all *n* vertices of the mesh and 3 · 4 · *f* bytes to represent the list of indices to edges of each triangle. To represent every edge that is represented once by the indices to the two vertices in the vertex list and the two triangles to which the edge belongs requires 4 · 2 · *e* + 4 · 2 · *e* = 24*f* bytes. In this case, the total amount of memory required to represent a mesh with *f* triangles with this structure equals 42*f* bytes.

Section 2 also described several known structures to provide fast access to topological adjacency and incidence data. It is not our purpose to extensively proove the storage cost for those structures.  Table 3 compares these data structures in terms of storage cost for a triangular mesh with *n* vertices and *f* ≈ 2*n* triangles. The “bytes/triangle” column presents the number of bytes needed for each triangle in memory. The variable *k* is the vertex degree or valency, that is, the number of edges incident at a given vertex.

The pointers to a vertex list data structure is the most compact of these structures since it only stores vertices and faces. It is used basically for visualization purposes.

For a graph *G* with *n* vertices and *e* edges, the sum of valencies of its vertices is *d* = ∑_*i*_^*e*^ *k*_*i*_ = 2*e*. Moreover, if *G* is induced from a triangular mesh 3*f* ≈ 2*e*, *n* − *d*/6 = 2 and *d* = 6(*n* − 2), the average vertex degree is approximately 6.

The new topological toolkit TopTri, presented in this paper, implements adjacency lists to represent the graph. For graphs obtained from triangular meshes, each vertex is adjacent to 6 vertices approximately. Assuming that each vertex requires 4 bytes to store its integer index, the total memory requirement for the graph is (6 · 4 + 4)*n* bytes. Because relations *n* − *e* + *f* = 2 and 3*f* ≈ 2*e* hold for these graphs, memory requirement is about 56 + 14*f*. Thus, for large values of *f* (*f* ≫ 56), the structure takes approximately 14 bytes for each triangle.

Table 3 presents only the topological memory cost for the TopTri since it is a toolkit for the topological data that can be used in combination with any geometric representation. Therefore, if the client application of TopTri uses the pointers to a vertex list to represent the geometry, which is the most concise representation, there is a total cost of 32 bytes for each triangle to store the geometric and topological data, which compares favorably with other known data structures.

## 6. Applications in a Web3D Server

In this section, we present some examples of functionalities that we have implemented using the topological framework TopTri installed in the Web3D server for the SIPCLIP project (http://193.136.227.170/sipclip/web3d.php). This project aims to provide information on past regional climates. This information is based on the analysis of cave speleothems, which are useful records for paleoclimatic reconstruction. These examples are built efficiently taking advantage of the topological information available from the TopTri library enabling their interactive use in a web browser.

**Figure 2:**
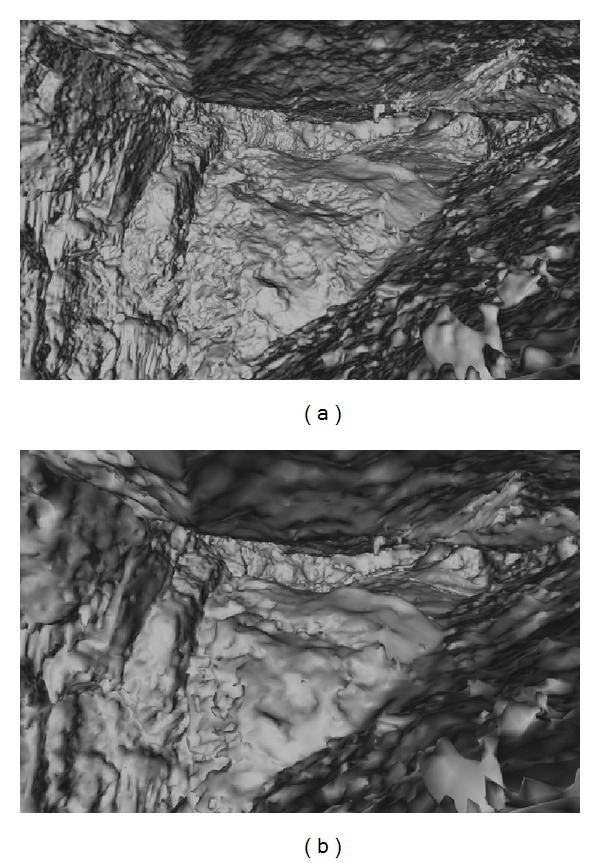
The 3D model of the cave chamber with 10 038 522 triangles (a) and its simplification after decimation process with 249 934 triangles for Web3D (b).

The 3D model of a cave chamber was built from a point cloud with about 45 million points obtained from a terrestrial laser scan survey. In order to select a 3D mesh with a reasonable size that does not compromise performance in the Web, we simplified the original cave chamber mesh with 10 038 522 triangles (Figure 2(a)), executing mesh decimation operations, to build a new 3D mesh model of the cave chamber with 249 934 triangles (Figure 2(b)) [21].

For the 3D mesh simplification we used the MeshLab multiedge decimation function called* Quadratic Edge Collapse Decimation*. This function removes the multiedge mesh together with its relative triangles and then connects the adjacent vertices to the new vertex [22].

Three simplifications were generated from the original cave chamber 3D mesh model.  Table 4 presents the number of triangular faces and the size of each simplified 3D mesh file in X3D format. Tests of download time were performed in* localhost* environment. Note that waiting times varying between 7 seconds and 60 seconds were measured. All the running times presented in this paper were obtained using a desktop computer with an Intel Core i7 3.40 GHz, 8 GB of memory RAM, and a NVIDIA Quadro 4000 graphic card (with 2 GB of dedicated memory).

### 6.1. Local Minima

In the following paragraphs we present the results obtained from the implementation of an algorithm to find local minima.

Speleothems are cave mineral deposits, usually formed of calcite whose precipitation processes are mainly related to carbon dioxide levels in the cave percolation water. Stalactites are speleothems hanging from the cave roof that form where percolation water seeps, mainly along geomorphological features in the cave ceiling such as faults or diaclases that represent preferential plans for water dripping. The recognition and positioning of cave stalactites can therefore give some information on major hidden cave features responsible for cave geomorphology. Stalagmites are speleothems that grow upward from the cave floor. They are therefore the complement of stalactites.

Stalactite extremities correspond to local minima in the 3D mesh. A local minimum in the 3D mesh surface is one vertex *v* of the triangular mesh such that its *z*-coordinate is smaller than the *z*-coordinates of all adjacent vertices of *v*. This local minimum is a stalactite extremity when its normal vector $\vec{n}=(0,0,n_{z})$ and *n*_*z*_ < 0.

**Figure 3:**
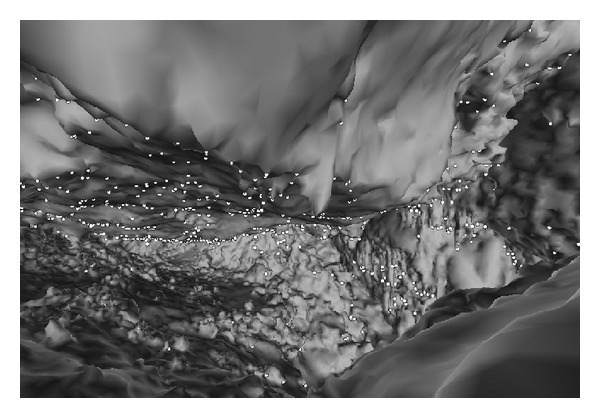
Web visualization of the cave chamber 3D mesh model with 249 934 triangles and local minima (stalactites extremities) as white points.

An algorithm to find local minima requires adjacency information. This was implemented using the adjacency information that we can get from the TopTri toolkit. For the graph *G* = (*V*, *E*), defined from the 3D mesh; Algorithm 2 returns a list of vertices such that their *z*-coordinate is smaller or equal to the *z*-coordinates of all adjacent vertices and the vector normal to the surface is downward oriented. The approach implemented finds all the local minima for the cave chamber in 80 milliseconds.  Figure 3 shows the interactive visualization of the 3D model of the cave chamber in a Web browser and the stalactite extremities presented as white points.

### 6.2. Stalactites

**Figure 4:**
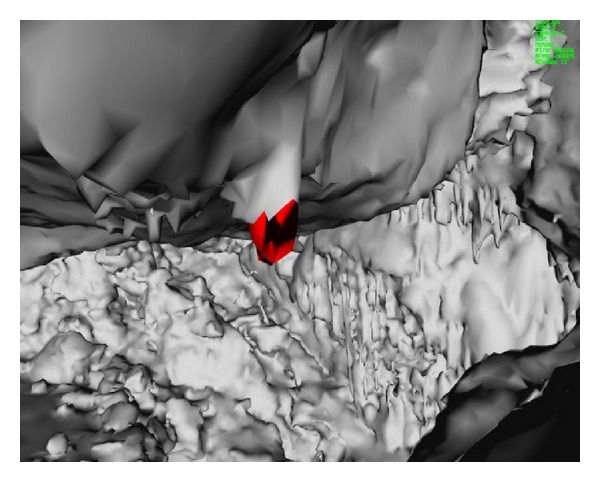
Stalactite selected interactively by the user in the Web3D browser.

As already mentioned, studying cave stalactites can be important. For this purpose, we also implemented a tool that enables the user to select one point of a stalactite and determine automatically the surrounding region of the stalactite. This is important for the determination of properties such as the surface area of the stalactite. This is implemented with Algorithm 3 that determines all the triangles of the stalactite using adjacency information available from the topological TopTri toolkit.  Figure 4 is an image of a stalactite with 96 triangles, area of 99.6 dm^2^, and bounding box dimensions of 16.3 cm × 15.9 cm × 24.1 cm, determined in 26 miliseconds with the previous algorithm.

### 6.3. Contour Lines

Contour lines on nonflat surfaces, also known as contours, are lines with all points at the same elevation. For a 3D mesh, if the *z*_0_ elevation contour line intersects an edge of the model, then the contour line has two segments that lie on the triangles *T*_1_ and *T*_2_ incident on that edge.

Providing the graph *G* = (*V*, *E*) and the elevation *z*_0_, Algorithm 4 returns the collection of the segments of the polyline *C*_*z*_0__ which represent the contour at elevation *z*_0_ of the 3D mesh.

This algorithm may run in linear time with respect to the number of the edges of the graph. It was used to compute a collection of contours with predefined equidistant between consecutive lines.

**Figure 5:**
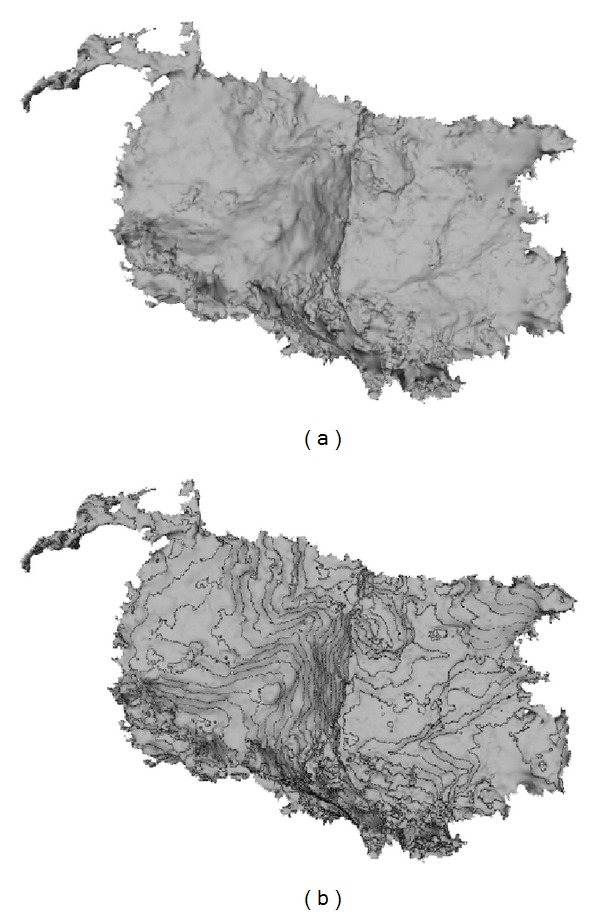
Visualization of the Web3D cave chamber model with 249 934 triangles from a top view perspective (a); and the same 3D mesh with 15 cm equidistant contour lines (b). Note that with the contours information the model highlights the cave relief and preferred alignments.

Contours provide rich information about morphology of the cave chamber surface. They help the identification of smooth regions, high gradient surfaces, and orientation of specific alignments.  Figure 5 shows the ceiling surface of the cave model from the top view without and with superimposed 15 cm equidistant contour lines (Figures 5(a) and 5(b), resp.), where those features can be easily recognized.

## 7. Evaluation of the Topological Framework

This section describes several tests to evaluate the performance of the novel topological framework TopTri presented in this paper.

**Figure 6:**
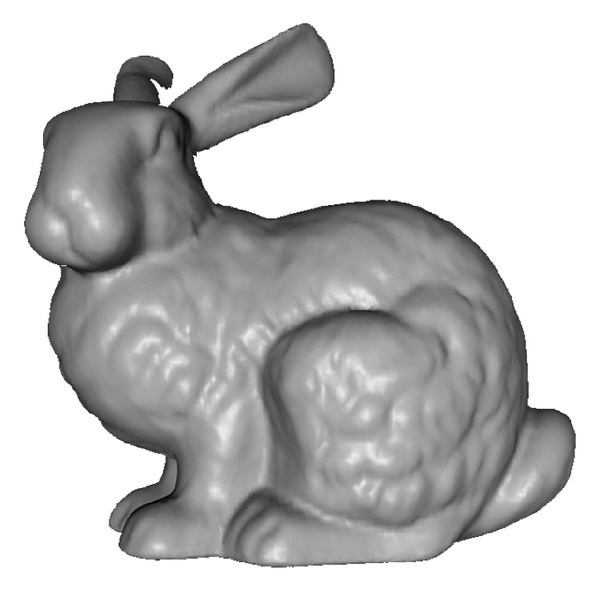
Bunny 3D mesh model.

In our experiments, we used the model of the bunny (http://graphics.stanford.edu/data/3Dscanrep/) from the University of Stanford (Figure 6), the model of the cave chamber for the Web3D (Figure 2(b)), and the original model of the cave chamber (Figure 2(a)) from the SIPCLIP project (PTDC/AAC-CLI/100916/2008—Temperature, precipitation regime and soil conditions in Southwestern Iberian Peninsula under a warmer climate—Insight from the past (http://193.136.227.170/sipclip)) [23].

Table 5 presents the complexity of these models. It can be seen that the model with the lowest complexity in terms of the number of triangles is the bunny with 69 451 triangles. The model of the cave chamber for the Web3D has 249 934 triangles and the original model of the cave chamber has 10 038 522 triangles. The original 3D model of the cave chamber was built from a point cloud with about 45 million points obtained from a terrestrial laser scan survey [21].

It can also be seen that the number of triangles *f* is about twice the number *n* of vertices (*f* ≈ 2*n*) as expected. The bunny, the Web3D, and the original cave chamber are stored in X3D format files of sizes 4.3 MB, 14.6 MB, and 520 MB, respectively. With these three models of different complexities we can evaluate the toolkit TopTri performance.

Topological relationship queries involve adjacency lists. It is known that the average of vertex degrees is approximately 6 for manifold triangular meshes. In addition to this result, we can see from Table 6 that six is the vertex degree most common in the three models and the number of vertices with degree greater or equal to 9 is less than 5 percent.

Table 7 presents the performance of the topological framework tested with the bunny, Web3D cave, and the original cave chamber models.

The first line shows the time to load the geometric model from the disk to main memory. It can be seen that the time to create the topological model ranges from 166 milliseconds to 24 seconds for the larger model with about 10 millions of triangles. These are acceptable values, even for the larger model. It is also seen that it always takes less time to create the topological model than to load the geometric model to memory.

Table 7 also presents the time to make adjacent and incident queries to the topological models. As the time to make a single query is so reduced, we decided to present in Table 7 the time to make 10 000 random queries. In this way, making 10 000 queries to get adjacent vertices to a given vertex are executed at most in 15 milliseconds for the original cave chamber model. The most time consuming operation is the query* vGetIncidentFaces* to get incident faces given a vertex as it can take about 71 milliseconds to make the ten thousand queries for the cave chamber.

Table 7 also confirms that to make an explicit topological relation query like* vGetAdjacentVertices*, which is retrieved in a single query, is faster than making an implicit topological query like* vGetIncidentFaces* that requires two or more queries. For this reason, the two explicit topological queries* vGetAdjacentVertices* and* vGetIncidentEdges* are the fastest.

Furthermore, from Table 7 we can also see that the time to make 10 000 queries does not change significantly with the complexity of the model. The cave chamber model has about 10 million triangles and the bunny model has about 70 thousand triangles but it takes 71 milliseconds and 64 milliseconds, respectively, to make ten thousand* vGetIncidentFaces* queries. This is explained since making searches in dictionaries in the Python programming language is of complexity *O*(1).

Nevertheless, memory usage to store the graph using these data structures result is greater than the estimated value of 14 bytes for each triangle. Measurements of memory allocated to the graph structure show that bunny takes about 90 bytes/triangle whereas Web3D cave and original cave chamber less than 82 bytes/triangle. This extra memory is related with the internal representation of above data structures and the fact that tests were performed in a 64 bits system.

The time needed to find all the local minima for the Web3D cave chamber model and for the original cave chamber model is 80 milliseconds and 3 seconds, respectively. The approach implemented finds all the local minima for a large model like the original cave chamber with about 10 million triangles.

## 8. Conclusions

This paper presents a free framework for 3D Web servers that builds a topological model for triangular manifold or nonmanifold meshes. Applications can use available methods to get adjacent and incidence information of vertices, edges, and faces.

This framework was implemented in Python (version 2.7). Although it is an interpreter language, results show that performance achieved makes the toolkit useful also for real-time applications. Furthermore, this programming language allows TopTri toolkit to be used in the server side for Web3D applications.

The framework described in this paper builds a topological model implementing a graph created from the triangle geometry mesh. In this way, client applications of the TopTri toolkit have access to adjacency and incidence information of vertices, edges, and faces.

Results show that getting adjacency and incidence information is very fast and at approximately constant time, which makes the TopTri toolkit scalable and appropriate for real-time applications.

It is shown in this paper that the memory cost per triangle to store the topological data compares favorable with other structures.

This paper also presents the use of the framework to the study of karsts and their geomorphological structures. We implemented algorithms to find local minima, isolines, and identify stalactites in a cave chamber in a Web browser.

## Figures and Tables

**Algorithm 1:**
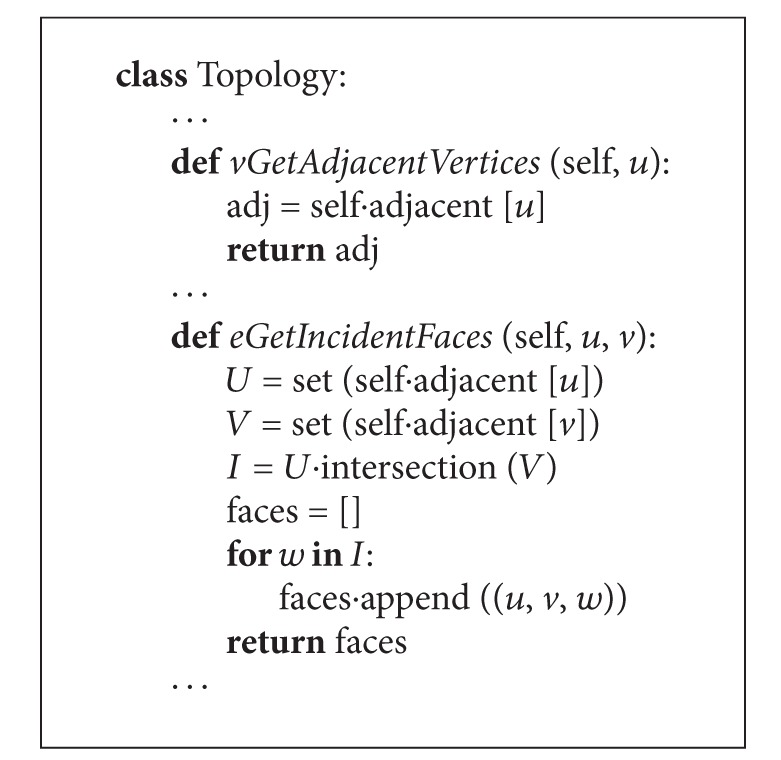
Implementation of methods *vGetAdjacentVertices *and* eGetIncidentFaces. *

**Algorithm 2:**
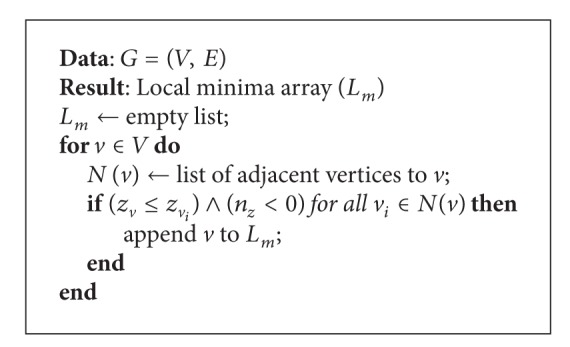
Local minima algorithm.

**Algorithm 3:**
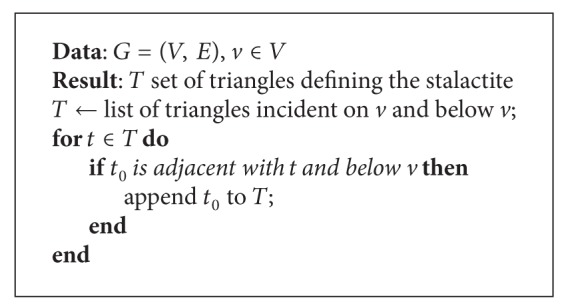
Algorithm to determine stalactites.

**Algorithm 4:**
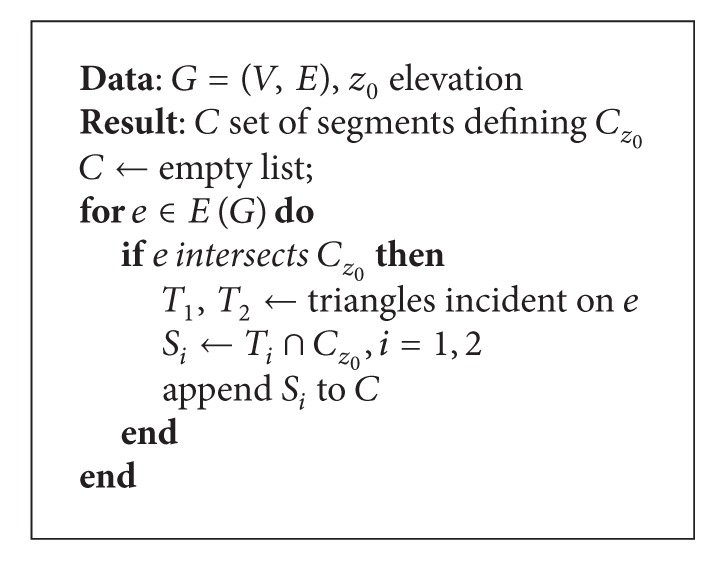
Contour lines algorithm.

**Table 1:** List of the implemented methods in TopTri toolkit for getting topological relationships for vertices, edges, and cycles of length 3.

Methods	Input	Output
*vGetAdjacentVertices *	Vertex	Vertices list
* vGetIncidentEdges *	Vertex	Edges list
* vGetIncidentFaces *	Vertex	3-cycle list
* eGetAdjacentEdges *	Edge	Edges list
* eGetIncidentFaces *	Edge	3-cycle list
* fGetAdjacentFaces *	3-cycle	3-cycle list

**Table 2:** Classification of explicit and implicit topological methods implemented in TopTri toolkit.

Methods	Property	Classif.
*vGetAdjacentVertices *	Property 1(a)	Explicit
* vGetIncidentEdges *	Property 1(b)	Explicit
* vGetIncidentFaces *	Property 1(c)	Implicit
* eGetAdjacentEdges *	Property 2(a)	Implicit
* eGetIncidentFaces *	Property 2(b)	Implicit
* fGetAdjacentFaces *	Property 3	Implicit

**Table 3:** Memory cost per triangle for the representation of geometry and topology.

Data structure	Bytes/triangle
Explicit	36
Pointers to a vertex list	18
Pointers to an edge list	42
Winged-Edge	60
Half-Edge	46
Radial-Edge	56
Adjacency and Incidence Framework	29 + 2*k*
Directed-Edge	44
Tri-Edge	35
Progessive Meshes	33
Progessive Simplicial Complexes	37 + 2*k*
Topological toolkit (TopTri)	14

**Table 4:** Number of triangles, X3D file size, and download time of three meshes generated after different decimation based on the original cave chamber model with 10 038 522 faces.

Decimation (%)	# Triangles	File size (MB)	Download time (s)
90	998 856	60.2	60
95	499 486	29.8	19
97	249 934	14.6	7

**Table 5:** Complexity in terms of the number of vertices, faces, and the X3D file size for the bunny, the Web3D, and the original cave chamber models.

	Bunny	Web3D cave	Original cave
# Vertices	35 947	126 921	5 021 214
# Triangles	69 451	249 934	10 038 522
File size	4.3 MB	14.6 MB	520 MB

**Table 6:** Frequency of vertex degrees for the tested models.

Degree	Bunny	Web3D cave	Original cave
≤3	0.12%	1.95%	0.02%
4	1.32%	9.00%	14.02%
5	11.33%	25.29%	23.29%
6	75.12%	31.82%	31.37%
7	11.16%	21.23%	16.91%
8	0.86%	8.25%	10.16%
≥9	0.07%	2.46%	4.22%

**Table 7:** Time to create topological model and to make 10 thousand adjacency and incidence random queries in the 3 models.

	Bunny	Web3D cave	Original cave
Time to load geometric model	177 ms	694 ms	32 s
Time to create topological model	166 ms	585 ms	24 s
*vGetAdjacentVertices *	14 ms	14 ms	15 ms
* vGetIncidentEdges *	23 ms	25 ms	26 ms
* vGetIncidentFaces *	64 ms	68 ms	71 ms
* eGetAdjacentEdges *	44 ms	49 ms	52 ms
* eGetIncidentFaces *	48 ms	51 ms	53 ms
* fGetAdjacentFaces *	61 ms	65 ms	67 ms
